# Condition-Based Maintenance with Reinforcement Learning for Dry Gas Pipeline Subject to Internal Corrosion

**DOI:** 10.3390/s20195708

**Published:** 2020-10-07

**Authors:** Zahra Mahmoodzadeh, Keo-Yuan Wu, Enrique Lopez Droguett, Ali Mosleh

**Affiliations:** 1 Department of Electrical and Computer Engineering and the B. John Garrick Institute for the Risk Sciences, University of California, Los Angeles–UCLA, Los Angeles, CA 90095, USA; zmah@g.ucla.edu; 2 Department of Materials Science and Engineering and the B. John Garrick Institute for the Risk Sciences, University of California, Los Angeles–UCLA, Los Angeles, CA 90095, USA; keoyuan0703@ucla.edu; 3 Department of Mechanical Engineering, University of Chile, Santiago 8320000, Chile; elopezdroguett@ing.uchile.cl

**Keywords:** dry gas pipelines, internal corrosion, condition-based maintenance, reinforcement learning

## Abstract

Gas pipeline systems are one of the largest energy infrastructures in the world and are known to be very efficient and reliable. However, this does not mean they are prone to no risk. Corrosion is a significant problem in gas pipelines that imposes large risks such as ruptures and leakage to the environment and the pipeline system. Therefore, various maintenance actions are performed routinely to ensure the integrity of the pipelines. The costs of the corrosion-related maintenance actions are a significant portion of the pipeline’s operation and maintenance costs, and minimizing this large cost is a highly compelling subject that has been addressed by many studies. In this paper, we investigate the benefits of applying reinforcement learning (RL) techniques to the corrosion-related maintenance management of dry gas pipelines. We first address the rising need for a simulated testbed by proposing a test bench that models corrosion degradation while interacting with the maintenance decision-maker within the RL environment. Second, we propose a condition-based maintenance management approach that leverages a data-driven RL decision-making methodology. An RL maintenance scheduler is applied to the proposed test bench, and the results show that applying the proposed condition-based maintenance management technique can reduce up to 58% of the maintenance costs compared to a periodic maintenance policy while securing pipeline reliability.

## 1. Introduction

The natural gas pipelines network transports natural gas from the wellhead to the customers throughout the USA. Corrosion is a very severe and costly issue in natural gas pipelines. It is specified as the gradual reduction in the pipeline wall thickness that might lead to substantial environmental and economic consequences as a result of catastrophic events, including leaks and ruptures. Therefore, pipeline integrity management has become highly essential [1]. Pipeline integrity management is composed of the pipelines’ inspection, repair, and maintenance with the scope of preventing failures and ensuring the safety of the pipelines during their lifecycles [2].

We are living in the era where the Internet of Things (IoT) is taking control over many tasks previously thought complicated, including asset integrity management [3]. The importance of pipeline integrity management leads to many investments and initiatives in implementing the IoT on the pipeline systems with the hope of reducing unnecessary costs and expenses. The immediate outcome of IoT implementation on the pipeline is an extensive monitoring system that can provide accurate, online information about various aspects and parameters of the pipeline. This accurate abundant data applies data-driven techniques for decision-making in gas pipeline integrity management [4].

Integrity management is intrinsically a sequential decision-making problem in which decisions have persistent dynamic effects; i.e., maintenance decisions not only affect the current condition of the asset, but their traces remain throughout the rest of the asset’s life. Reinforcement Learning (RL) is an area of Artificial Intelligence (AI) concerned with data-driven modeling and solving sequential decision-making problems under uncertainty. It has three distinguishing characteristics that make it well suited to formulate the asset integrity management problem:

- RL algorithms can learn from historical and online data [5,6]: as IoT monitoring and operating systems continue to grow, more data from the asset condition will be accessible which provides an unprecedented opportunity to generate tools that may learn optimal condition-based maintenance from the data.
- RL can deal with delayed effects [5,6]: most maintenance actions have highly delayed indications of efficiency which makes the optimal integrity management a very hard problem to deal with. RL is to some extent capable of dealing with delayed consequences. Therefore, it gives us the proper tool to handle the delayed effects of actions.
- RL algorithms are designed to interact and learn in a stochastic environment [5], [6]: oftentimes, the assets are exposed to unpredictable operating and environmental conditions that lead to high uncertainty in the effectiveness of the maintenance actions. RL is known to be capable of taking into account the high-uncertainty environment.

RL techniques for asset integrity management have been attracting more and more attention in recent years. Many studies have shown that an RL-based policy exhibits a superior performance than periodic or corrective policies. For example, in the manufacturing industry, Xanthopoulos et al. [7] employed a policy-based Q-learning algorithm to find the optimal joint production/maintenance policy while minimizing the inventory level of a production/inventory system. Wei and Qi [8] also applied a policy-based Q-learning algorithm to study the optimal actions including produce, idle, and maintenance, to maximize the average reward in a two machines-one buffer system. In addition to manufacturing systems, RL techniques were also applied to some large-scale and complex systems for operation and maintenance management. Aissani et al. [9] proposed a dynamic scheduling of maintenance plans on an operating oil refinery by state-action-reward-state-action (Sarsa) algorithm to maximize the system availability and production efficiency. Barde et al. [10] took multiple independent components in a military truck into account and found the optimal maintenance time for each component to minimize the system downtime by Monte Carlo RL. Compare et al. [11] developed a framework by a Sarsa algorithm to find the best part flow management strategy of gas turbines consisting of repair and purchase actions in order to minimize the cost. In a more recent study, Bellani et al. [12] proposed a Prognostics and Health Management (PHM) framework based on sequential decision-making and RL that, compared with the current practice of PHM, allows consideration of the maintenance and operation decisions’ influence on predicting the degradation.

The corrosion-related maintenance and operation of oil and gas pipelines in the United States account for a large portion, about 80%, of annual corrosion-related costs, which is estimated to be 7 billion US dollars according to the National Association of Corrosion Engineers (NACE) [13]. This substantial economic trace of corrosion demands more investigations into minimizing the corrosion-related costs. Inspired by the abovementioned real-world applications of RL techniques on asset integrity management, this research aims at (a) demonstrating how to apply RL techniques to the maintenance scheduling of a dry natural gas pipeline subject to internal corrosion, and (b) investigating the possible reduction in the corrosion-related costs through the deployment of RL algorithms.

The great recent achievements of RL, or more particularly deep reinforcement learning, are to some extent owed to the notable opensource testbeds that became available to the RL research community, namely OpenAI Gym [14] and the arcade learning environment [15]. For example, Wang et al. [16] proposed an algorithm that achieved a superhuman performance in 57 Atari games that is implemented and trained within the arcade learning environment. Despite all the great achievements of RL in video and Atari games, it has minimal success in safety-critical applications such as asset integrity management. One reason is the lack of a trustable test bench that could evaluate the performance of the AI approaches to real-world problems. In this research, we address this issue in pipeline maintenance management by proposing a test bench that could interact with any maintenance decision-maker, including RL algorithms.

Furthermore, we propose an autonomous maintenance scheduler based on RL techniques that interacts and communicates with the test bench to achieve an optimal maintenance policy. The proposed decision-maker integrates the health state data of a physical asset with a data-driven, model-free RL algorithm to ensure the asset’s integrity, as measured by the desired level of reliability, while minimizing the maintenance costs and extending the life of the asset. Therefore, the approach is condition-based because it leverages upon the asset’s health state condition information, and it is autonomous because it applies RL techniques and algorithms to come up with the optimal time and action of the maintenance. In summary, the motivations of this study are:

1. Investigating the capabilities of RL as a mathematical tool to digest the abundant data that are collected and stored from the pipelines over the years.
2. Filling the gap between the trending RL developments and pipeline maintenance decision-making by introducing a test bench for pipeline maintenance and an RL agent interaction. The test bench mimics the challenges of pipeline maintenance management; however, it intentionally reduces the problem complexity.

As there is limited research on using the RL technique for the maintenance optimization of corroded gas pipelines, this research can be regarded as a benchmark for interaction evaluation between a maintenance decision-maker and a corroded gas pipeline that could facilitate future research on the effectiveness of various RL techniques in condition-based corrosion maintenance management. The main contributions of this research are:

1. Proposing and implementing a test bench for interaction evaluations between a maintenance decision-maker and a corrosive pipeline in terms of the system reliability level and the cost effectiveness of the maintenance decisions.
2. Proposing and implementing a condition-based maintenance management regime that leverages RL techniques to achieve a superior performance (in the proposed test bench) compared to the existing maintenance management practices.
3. Implementing a corrosion prediction model for a gas pipeline that could adjust to include the influence of multiple concurrent maintenance actions.

The remainder of the paper is structured as follows. The next section elaborates on the design and implementation details of the proposed test bench. It first states the assumptions that are made in the test bench development, and then it goes through the details of the test bench components. Afterward, the proposed RL condition-based maintenance scheduler is presented in Section 3. Section 3 presents some RL preliminaries and then demonstrates the heart of the RL maintenance scheduler, which is a Q-learning-based algorithm. Following that, Section 4 explains our evaluation methodology, which is how the RL maintenance scheduler’s performance is assessed and compared with the current widely used industry maintenance strategy, periodic maintenance practice. Then, the results of applying the proposed methodology to the pipeline corrosion maintenance management are shown in Section 5—Results and Discussion. Finally, some concluding remarks and possible future work for improvement are provided in Section 6—Conclusions.

## 2. The Test Bench

As stated in the introduction, the proposed condition-based maintenance scheduler is based on model-free RL. This class of RL algorithms is completely data-driven and does not need any knowledge about the system. In contrast with the model-based approaches, e.g., dynamic programing, model-free approaches do not require an accurate state transition function but still should be provided with observations and reward signals from the system. In this research, we propose and implement a test bench that is needful to evaluate the performance of any decision-making entity prior to performing direct interactions with a real pipeline system. This test bench:

1. Simulates the internal corrosion of the pipeline benefitting from a corrosion model that is adjustable to multiple concurrent maintenance actions and capable of simulating the corrosion with daily precision.
2. Simulates the pipeline environmental and operating parameters with daily precision.
3. Provides interaction protocols between the maintenance decision-maker and the pipeline’s corrosion simulator.
4. Approximates the cost associated to the maintenance actions and the pipe health state.

It is worth emphasizing that the decision-maker does not have access or knowledge about the internal behavior of the corrosion model and treats that as a black box.

Prior to getting into the details of the test bench, we will state the assumptions that are made in the test bench implementation.

1. The system is one section of the pipeline which is presumed to be new at time t = 0, and a corrosion defect gradually develops after that depending on the time-varying operating conditions.
2. It is assumed that the pipeline section suffers from only one corrosion defect at each time.
3. The pipeline section is assumed to corrode by two types of corrosion, namely pitting and uniform corrosions. To aggregate the two corrosion processes, a linear combination of the two is considered, as suggested by Wu and Castro [17]. In this case, when the combined degradation reaches the threshold values, the pipeline system fails either due to leak or rupture.
4. Two types of maintenance are considered, namely, preventive maintenance and a replacement of the system. The preventive maintenance includes a batch corrosion inhibitor, internal coating, and pigging. The replacement renews the system completely.
5. Determining the in line inspection (ILI) interval is always a challenging problem to pipeline operators which depends on the conditions of the pipeline systems [18]. In this paper, we simplify the problem and focus on the implementation of the RL techniques by assuming that an RL maintenance scheduler receives signals from the health state of the pipeline through monthly inspections. Then, it can decide either to do nothing, carry out one of the preventive maintenance actions or replace the corroded segment of the pipeline The scheduler’s maintenance order is disclosed to the system at the beginning of each month.
6. Inspection and maintenance times are neglected.

The following subsections describe the implementation details of the proposed test bench. First, an overview of various components of the test bench and their interactions is presented, then the details of each particular component is further elaborated.

### 2.1. Components of the Test Bench

The test bench is composed of three components. First, a set of environmental and operational parameters are simulated that mimic the daily variation of a real pipeline’s environmental and operational parameters. Afterward, the parameters are fed into a pipeline model along with the order of the month’s maintenance action. Then, the pipeline corrosion model adjusts itself to the received maintenance order and then simulates the corrosion progress over a month-long period. At the end of the month, the total corrosion depth and length is inputted to the pipeline reliability model that simulates the failure events, namely leak and rupture. Eventually, a cost approximator estimates the month’s expenditure according to the existence of any failure as well as any maintenance actions.

**Figure 1:**
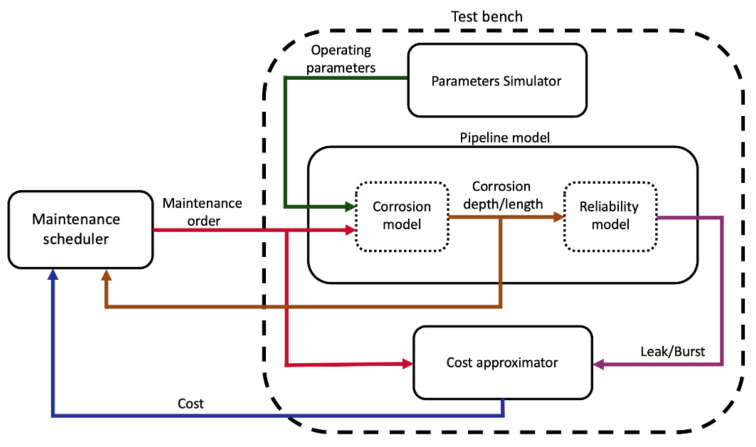
The proposed test bench for interaction evaluation between a maintenance decision-maker and a corrosive pipeline.

Figure 1 shows an overview of the proposed test bench. It should be emphasized that the maintenance schedular is not part of the test bench. It is a separate entity that interacts with the test bench by reading the end of the month corrosion condition and the cost, and then instructing a maintenance order for the next month. The choice of the maintenance actions is limited to the set of actions that the corrosion model could adjust to them.

### 2.2. The Environmental and Operational Parameters Simulator

The complex operating condition of the pipeline system is simulated by modeling a variety of environmental parameters. Most common environmental parameters that are related to the pipeline corrosion include temperature (*Temp*), total pressure (*P*), partial pressure of CO_2_ (*pCO_2_*), partial pressure of H_2_S (*pH_2_S*), flow velocity (*V*), pH value (*pH*), chloride ion (*Cl^−^*) concentration, and sulphate ion (*SO_4_^2−^*) concentration, and the presence of solids ($R_{solid}$). The environmental parameters are stochastic in nature; therefore, in order to take temporal variability into account, we used a Poisson Square Wave Process (PSWP) [19,20] to model them and generate different time-varying environmental parameters. In a PSWP, the number of pulses (Z) within a period of time (ΔT) follows a Poisson distribution with the probability mass function being:

(1) $$P\left(Z=z|\lambda \right)={\left(\lambda \Delta T\right)}^{z}\exp\left(-\lambda \Delta T\right)/z!$$

where λ is the mean occurrence rate per unit time (= 1 per day in this paper); ΔT follows the exponential distribution with the mean duration to be 1/λ. The magnitude of each environmental parameter is described by a specified distribution such as uniform, normal, and lognormal distributions followed by a coefficient of variability (COV). The values are based on several studies [21,22] and the details are shown in Table 1. The simulated environmental parameters are the inputs of the corrosion model to quantify the system degradation by internal corrosion, which will be introduced in the following subsection, on a daily granularity.

### 2.3. The Pipeline Model

The simulated system is a dry natural gas pipeline, which is 1.6 km in length, has an outer diameter of 509 mm, and inner diameter of 492 mm. The pipeline is considered to be used for transporting natural gas (i.e., CH_4_) with a small amount of corrosive gases (i.e., CO_2_ and H_2_S) and elements (i.e., Cl^−^). The degradation is the result of internal corrosion, including uniform and pitting corrosions. Degradation is a function of the operating conditions which are considered and simulated as time-varying parameters. When the linear combination of the two degradation processes reaches the threshold values, the pipeline fails either due to leak or rupture. In summary, the pipeline model takes the environmental parameters as inputs and outputs two Boolean signals representing whether the pipeline fails by either leak or rupture. The pipe model consists of a corrosion model and a reliability model, each of which will be described in the following subsections.

#### 2.3.1. Corrosion Model

**Figure 2:**
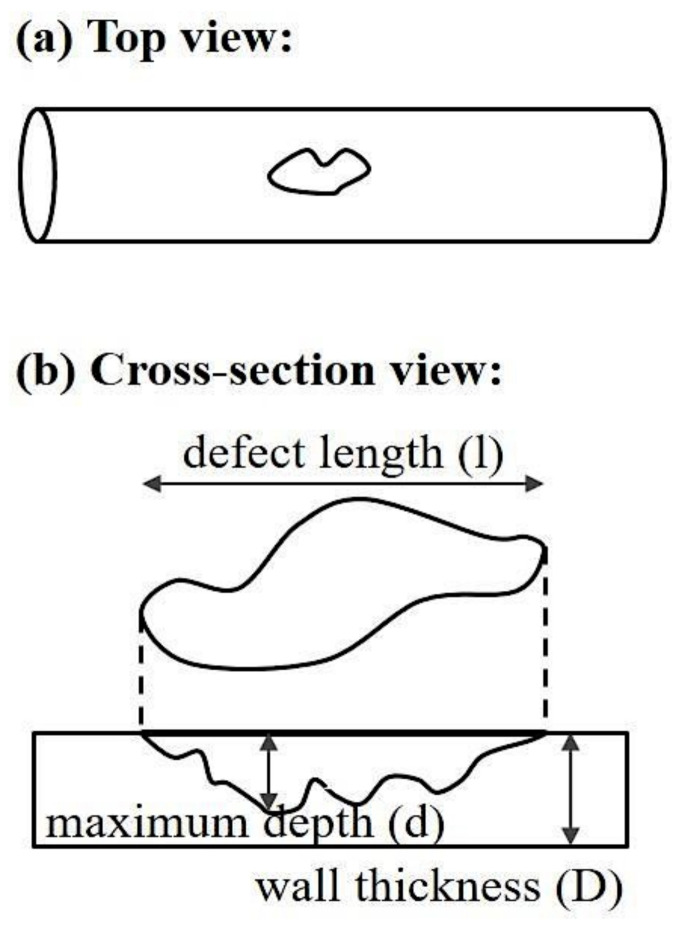
A rectangular-like corrosion defect in the pipeline in (**a**) top view and (**b**) cross-section view.

The pipeline system degrades as a result of internal corrosion including uniform and pitting corrosion. The corrosion defect is assumed to have rectangular-like shape as shown in Figure 2 and can be described by corrosion depth (d) and corrosion length (l).

To simulate the overall degradation by a multiple degradation process (i.e., uniform and pitting corrosion), a linear combination method [17] was adopted. The expression of the overall degradation is given by:

(2) $$Y\left(t\right)=W_{UC}X_{UC}\left(t\right)+W_{LC}X_{LC}\left(t\right)$$

where $Y\left(t\right)$ is the overall corrosion degradation over time in depth and length (with $Y\left(t\right)\;\epsilon \;\{d\left(t\right),l(t)\})$; $W_{UC}$ and $W_{lC}$ are the weight factors of uniform and pitting corrosion, respectively (with $W_{UC}\;\epsilon \;\left[0,1\right]\;and\;W_{LC}\;\epsilon \;\left[0,1\right])$; $X_{UC}$ and $X_{LC}$ represent the corrosion degradation over time in the depth and length of uniform and pitting corrosion, respectively (with $X_{UC}\left(t\right)\;\epsilon \;\left\{d_{UC}\left(t\right),\;l_{UC}\left(t\right)\right\}$, $X_{LC}\left(t\right)\;\epsilon \;\left\{d_{LC}\left(t\right),\;l_{LC}\left(t\right)\right\}$).

Corrosion depths over time by uniform corrosion ($d_{UC}\left(t\right)$) and pitting corrosion ($d_{LC}\left(t\right)$) are regarded as the accumulation effect of the daily corrosion rate, which refers to the daily granularity assumption of environmental parameters; therefore, they can be calculated as:

(3) $$d_{UC}\left(t\right)=\overset{K}{\underset{i=1}{\sum }}\frac{{CR}_{i}\left(t\right)}{365}$$

(4) $$d_{lC}\left(t\right)=\overset{K}{\underset{i=1}{\sum }}\frac{{PCR}_{i}\left(t\right)}{365}$$

where $CR_{i}\left(t\right)$ is the uniform corrosion rate over time; $PCR_{i}\left(t\right)$ is the pitting corrosion rate over time; K is the number of days.

**Figure 3:**
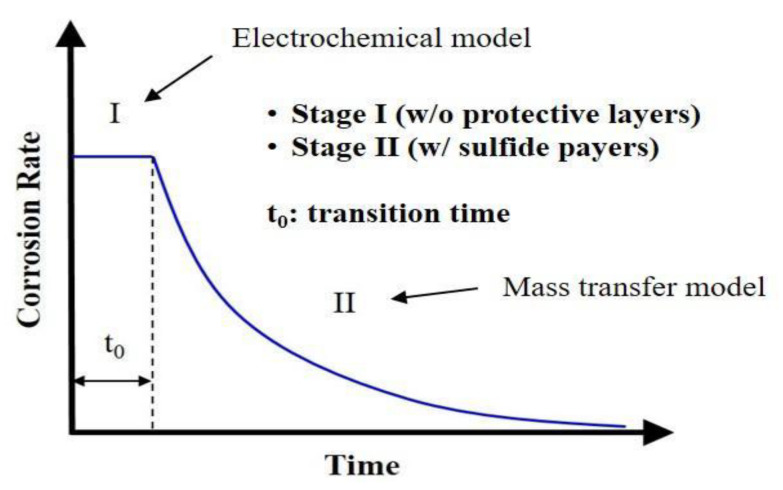
A schematic diagram of the two-stage model for uniform corrosion as a function of time (after [20]).

First of all, uniform corrosion rate is simulated by the two-stage model proposed by the authors of [20]. As uniform corrosion usually follows by the formation of protective layers, this predictive model describes the corrosion behavior in a phenomenological sense in terms of two stages depending on the formation of corrosion-protective layers. Figure 3 shows the concept of this model for uniform corrosion as a function of time. Specifically, Stage I simulates the corrosion behavior in the absence of protective layers using the electrochemical model, which involves the calculation of corrosion current densities for all electrochemical reactions (i.e., iron dissolution, hydrogen ion reduction, direct carbonic acid reduction, direct hydrogen sulfide reduction, and direct water reduction) that may take place in the CO_2_/H_2_S aqueous environment inside the pipeline.

The uniform corrosion rate at Stage I ($CR_{I}\left(t\right)$) is represented as:

(5) $${CR}_{I}\left(t\right)=\frac{i_{corr}M_{\mathrm{Fe}}}{\rho_{\mathrm{Fe}}2F}$$

where $i_{corr}$ is the corrosion current density in A/m^2^; $M_{\mathrm{Fe}}$ is the molar mass of iron (= 55.85 g/mol); $\rho_{\mathrm{Fe}}$ is the density of iron (= 7.86 g/cm^3^); F is the Faraday constant (= 96,500 C/mol).

Stage II simulates the corrosion behavior in the presence of protective layers using the mass transfer model. Due to the formation of mackinawite sulfide layers on the steel surface, several reactions may take place in the corrosive environment including the diffusion of corrosive species such as HS^−^, S^2−^, HCO_3_^2−^, CO_3_^2−^ from the bulk solution to the steel surface, release of dissolved iron sulfides such as Fe(HS)^+^, HS^−^ from the protective layers to the bulk solution, and the release of Fe^2+^ from the steel surface to the bulk solution. The corrosion rate is influenced by the fluxes of H_2_S, CO_2_, and H^+^ in which the thickness of mackinawite sulfide layers has a critical role in the level of corrosion. The uniform corrosion rate at Stage II ($CR_{II}\left(t\right)$) is represented as:

(6) $${CR}_{II}\left(t\right)=\frac{\phi_{H_{2}S}M_{\mathrm{Fe}}}{\rho_{\mathrm{Fe}}}+\frac{\phi_{{\mathrm{CO}}_{2}}M_{\mathrm{Fe}}}{\rho_{\mathrm{Fe}}}+\frac{\phi_{H^{+}}M_{\mathrm{Fe}}}{\rho_{\mathrm{Fe}}}$$

where $\phi_{H_{2}S}$, $\phi_{CO_{2}}$, and $\phi_{H^{+}}$ are the fluxes of H_2_S, CO_2_, and H^+^ in mol/(m^2^s). For more model details, readers are referred to [20].

**Figure 4:**
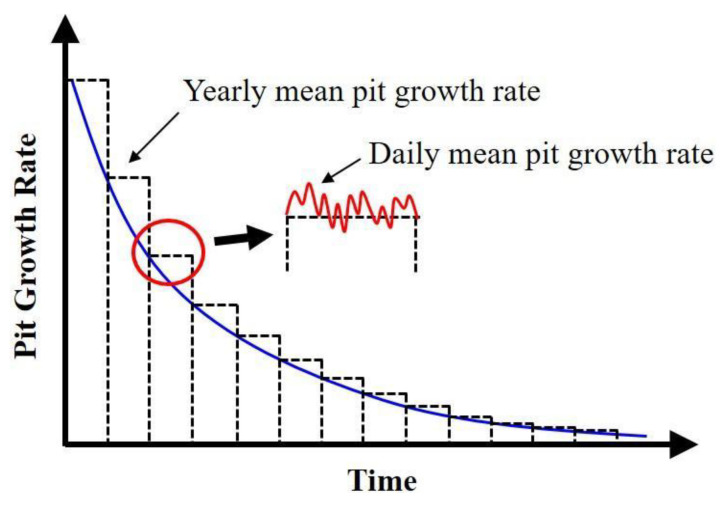
A schematic diagram of the model for pitting corrosion as a function of time.

On the other hand, the pitting corrosion rate is simulated by the Papavinasam model [23]. This model takes different environmental parameters and predicts the yearly mean pit growth rate given the constant operating condition. Each of these environmental parameters has its individual equation with respect to the pitting corrosion rate and they are integrated into a final corrosion rate equation:

*PCR_mean_* = {(−0.33 *θ_c_* + 55) + (0.51 *W*% + 12.13) + (0.19 *W_ss_* + 64) + (50 + 25 *R_solid_*) + (−0.081 *P* + 88) + (−0.54 + 67) + (−0.63 *pCO_2_* + 74) + (−0.013 *[SO_4_^2−^]* + 57) + (0.57 *Temp* + 20) + (−0.014 *[HCO_3_^−^]* + 81) + ( 0.0007 *[Cl^−^]* + 9.2) + *PCR_addition_*}/12 (7)

where *θ_c_* is the contact angle of oil in a water environment in degree; *W%* is the water production rate/(water + oil production rates) × 100; *W_ss_* is the wall shear stress in Pa; *R_solid_* is 1 if solids exist, otherwise it is 0; *Temp* is the temperature in °C; *P* is the total pressure in psi; $pH_{2}S$ is the partial pressure of H_2_S in psi; $pCO_{2}$ is partial pressure of CO_2_ in psi; *[SO_4_^2−^]* is the sulfate concentration in ppm; *[HCO_3_^−^]* is the bicarbonate concentration in ppm; *[Cl^−^]* is the chloride concentration in ppm; *PCR_addition_* is the mean pitting corrosion rate of these 11 individual pitting corrosion rates. Due to the consideration of daily-varying environmental parameters, this model is modified to a model that can predict the daily mean pit growth rate as a function of time. The schematic diagram of this model is shown in Figure 4.

Unlike the modeling of corrosion depth that have been studied for a long time, to the best of the authors’ knowledge, there are no widely accepted model to calculate corrosion length [24]. Therefore, we adopted the linear growth model [25] with an arbitrarily assumed corrosion length growth rate (LGR) for corrosion length simulation given the assumption that LGR is the same for uniform and pitting corrosion. Consequently, the corrosion length over time by uniform ($l_{UC}\left(t\right)$) and ($l_{LC}\left(t\right)$) can be represented as:

(8) $$l_{UC}\left(t\right)=l_{LC}\left(t\right)=\mathrm{LGR}\times \frac{t}{365}$$

The simulated overall corrosion depth $d\left(t\right)$ and length l(t) are inputs to the reliability model, which will be introduced in the following subsection.

#### 2.3.2. Reliability Model

When the corrosion degradation of the system exceeds the threshold values, the pipeline system fails either due to leak or rupture. We use a reliability model to determine whether the system fails. This reliability model is based on the load and strength interference technique by defining their limit state functions at a given corrosion defect. The limit state functions of leak ($g_{1}$) and rupture ($g_{2}$) can be written as:

(9) $$g_{1}=\Lambda -d_{max}$$

(10) $$g_{2}=P_{b}-P_{op}$$

where Λ is the corrosion allowance (i.e., 0.8 × wall thickness); $d_{max}$ is the maximum defect depth; $P_{b}$ is the burst pressure of the corroded pipeline; $P_{op}$ is the operating pressure.

Limit state functions show that a leak happens when the maximum defect depth is larger than the corrosion allowance ($g_{1}\leq 0$), while a rupture happens when the operating pressure is larger than the burst pressure ($g_{2}\leq 0$).

The $P_{b}$ of the pipeline is calculated by the ASME B31G standard [26] because it requires relatively less information of the defect geometry (i.e., only corrosion length and depth) and, therefore, it is easier to implement. In addition, calculations by this model in terms of the probability of failure fall in the safe domain, implying that they are not too conservative in their calculation of the probability of rupture compared to the other existing standards or models [27]. According to the ASME B31G standard, $P_{b}$ can be represented as:

(11) $$P_{b}=\sigma_{h}\frac{2w}{D}=\sigma_{f}\left[\frac{1-\frac{A}{A_{0}}}{1-\frac{A}{MA_{0}}}\right]\left(\frac{2w}{D}\right)=\left(1.1\sigma_{y}\right)\left(\frac{2w}{D}\right)\left[\frac{1-A/A_{0}}{1-\frac{A}{A_{0}}/M}\right]$$

where $\sigma_{h}$ is hoop stress; $\sigma_{f}$ is the flow stress; w is the pipe’s wall thickness; D is the pipe’s outer diameter; A is the surface area of the corrosion defect; $A_{0}$ is the original surface area of the pipe with $A/A_{0}=2d/3w$ where d is the depth of a corrosion defect; M is the Folias factor defined as:

(12) $$M=\sqrt{1+0.8{\left(\frac{l}{D}\right)}^{2}\left(\frac{D}{w}\right)}for\sqrt{0.8{\left(\frac{l}{D}\right)}^{2}\left(\frac{D}{w}\right)}\leq 4$$

(13) $$M=\infty \;\;for\;\;\sqrt{0.8{\left(\frac{l}{D}\right)}^{2}\left(\frac{D}{w}\right)}>4$$

where l is the length of a corrosion defect.

Given the limit state functions, the probability of failure with respect to each of these two failure modes can be determined by a Monte Carlo simulation [28]:

(14) $$P_{f}=\frac{N_{B}\left[g<0\right]}{N_{S}}$$

where $P_{f}$ is the probability of failure (with $P_{f}\;\epsilon \;\left\{P_{f,leak},\;P_{f,burst}\right\}$); g(x) is the limit state function (with $g\epsilon \left\{g_{1},\;g_{2}\right\}$), and $N_{S}$ is the number of simulations.

To simplify the problem as a first approximation, we assume that leaks and ruptures are independent; therefore, binomial distributions, $f\left(k,n,p\right)=p^{k}{\left(1-p\right)}^{n-k}$ where k is the number of success event; $N_{B}$ is the number of Bernoulli trials; p is the probability of success, which is used to determine whether the pipeline system fails. Specifically, two samples are driven from each Binomial distribution representing leak $f\left(1,2,P_{f,leak}\right)$. and rupture $f\left(1,2,P_{f,burst}\right)$, and if either one of the events happen, the pipeline is regarded as a fail.

#### 2.3.3. Adjustment of the Corrosion Model to Maintenance Actions

Two types of maintenance, namely preventive maintenance and replacement, are considered. Preventive maintenance includes a batch corrosion inhibitor, internal coating, and pigging, all of which are common for the mitigation of internal corrosion in dry natural gas pipelines [29]. It should be noted that although internal coating [30] is usually applied during the manufacturing process, it is not uncommon for pipeline operators to do coating repair if the coating is damaged due to corrosion [31]; therefore, internal coating is considered as one maintenance action here for comparison. Doing nothing is also an option to provide the agent with the flexibility of applying no maintenance action.

The corrosion model has daily granularity. However, the interaction of maintenance decision-maker and the environment happens on a monthly basis. On the first day of each month, the agent gets informed about the new state of the pipeline through monthly inspections and subsequently determines this month maintenance action and executes that action. Afterward, the pipeline model receives the action of the month and adapts itself accordingly. The details of the considered maintenance actions are shown in Table 2.

For asset management problems, failure intensity is a common metric used for quantifying the effect of repair models. For example, Doyen and Gaudoin [32] propose the reduction in intensity model to express the repair effect on the reduction in the system failure. Inspired by this paper, we propose a discount factor (df) ranging between zero and one to account for the influences of maintenance actions on the pipeline corrosion. The corrosion rate is multiplied by the discount factor to model the updated corrosion rate. Each maintenance action with a direct influence on the corrosion rate has a lifetime. The discount factor becomes 1 beyond the lifetime. The discount factors and the lifetime with respect to different maintenance actions are listed in Table 3.

According to Table 3, immediately following a batch corrosion inhibitor appliance, the corrosion rate would greatly lessen, and afterward gradually increase to its original value by an exponential function as stated in [34]. In addition, the effect of the coating appliance and the pigging is to set the corrosion rate to zero but with different time scales. The coating effect is more permanent and remains for a longer period of time; therefore, the corrosion rate would be set to zero for five years. However, following a pigging action the corrosion rate would be set to zero only for two weeks.

### 2.4. Development of the Cost Approximator

Defining the costs is a critical task in any decision-making problem because the reward/cost is the only mean by which the decision-maker can know the aptness of its actions. The goal of the maintenance decision-maker in the corrosion maintenance scheduling problem is to:

1. Avoid catastrophic failures, namely leakage and rupture.
2. Extend the life of the asset.
3. Reduce the maintenance costs.

To include these three goals in the reward function, we define three types of rewards as follows. Here, we use cost to address the negative reward, and the total reward after each month is the algebraic summation of these three values:

1. Cost of failure: the cost that is associated with the situations where the system has failed. This cost will be non-zero only when the pipeline enters a failure state. Immediately upon arrival of the pipeline in a failed state, a large cost will be emitted to the decision-maker to penalize its decision-making policy. This cost should be considerably larger than the other types of costs because of the massive hazard the failures imposed on the life of people and the pipeline’s surrounding environment. Besides, failures cause an interruption in the service that might have large economic consequences.
2. Life extension reward: the reward of delaying the failure. At the end of each month that the pipeline is not in one of the terminal states, namely failure and replacement, the agent receives a positive reward for extending the life of the pipeline for that month. This value is small in comparison to other types of cost.
3. Cost of maintenance: the cost that is associated with each maintenance action. The cost of the month’s maintenance action is emitted to the agent at the end of each month along with the failure delaying reward and the failure cost.

One of the challenges in designing the test bench is assigning values to the reward function since the cost-sensitive decision-making mechanisms highly rely on the cost values. The approach taken in this research is to assign values to the maintenance cost from generic information appertaining to the gas pipeline maintenance and then specify the remaining reward values with respect to the maintenance costs. It should be noted that the values assigned here could be updated for different application scenarios. In addition, at this stage, the problem is simplified by considering only a segment of the pipeline, which is 1-mile long. By doing this, we are able to ignore the spatial effect on our studied system.

Table 4 lists the values of the three types of reward functions considered in this study. Regarding the maintenance cost, first of all, “Do nothing” costs USD 0 in general if the cost of the inspection is neglected for all the other maintenance actions. Second, “Pigging” operation starts from the inlet to the outlet; therefore, the cost is determined based on the distance between the launcher and the receiver. Based on the data from pipeline operators, the cost is roughly USD 35,000/mile [35]. Third, “Batch-treatment of corrosion inhibitor” is to pump the corrosion inhibitors from the inlet, and the cost is determined by the volume of the pipeline. Here it is assumed that one-third of the tubing volume is needed for the inhibition efficiency to be over 90%, and the cost is estimated to be USD 130,000/mile [33]. In addition, “Internal coating” is a costly option as it requires the pipeline excavation. The cost is approximately USD 800,000/mile according to [36]. Finally, the cost of the replacement of a corroded pipeline varies on the basis of the pipeline size, which is hard to be accurately estimated, but in general it is more expensive than any rehabilitation actions. Here, we assign it to be USD 1,600,000/mile.

Two types of failure costs are considered in this research. The leak cost is assumed to be three times the replacement cost because a leaked pipeline needs to be replaced but the replacement follows with an unplanned interruption of the service. The rupture cost also includes the replacement cost but along with that, it has a significantly larger cost reflecting the highly catastrophic hazards it might lead to. The life extension reward per month is set to USD 70,000 based on the results of the sensitivity analysis that was performed on the value of the life extension reward in the evaluation section.

## 3. The RL Maintenance Scheduler

In this section, we first review the RL fundamentals in maintenance management, and then we present the methodology and the algorithm for the proposed RL-based maintenance decision-maker. Among various maintenance management strategies, this study focuses on the condition-based maintenance strategy that incorporates the costs into the decision-making process because they are shown to be more cost-effective over the pure risk-based and time-based strategies [37,38]. The goal of the scheduler is to find the best time and type of maintenance actions that optimize the maintenance-related costs while ensuring a high level of reliability in the pipeline.

### 3.1. Preliminaries

**Figure 5:**
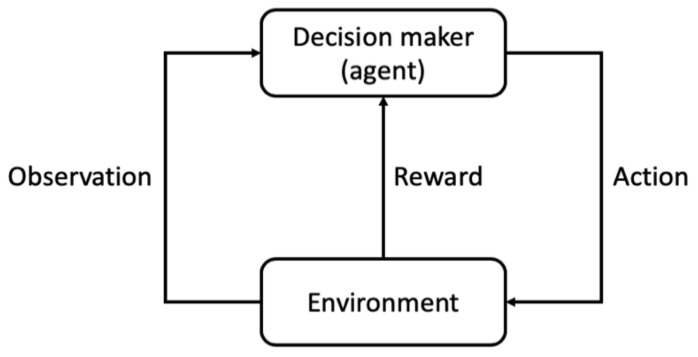
Reinforcement learning (RL) agent and the environment interaction.

In the general RL setting, a decision-maker (often called as agent) interacts with an uncertain environment to achieve a goal. The environment is influenced by the agent’s actions, and the agent can observe the influence through some observation signals as well as a reward signal. The reward signal should reflect the aptness of the previous actions, and the agent learns to improve and optimize its decisions by learning from the previous rewards. The underlying assumption in all RL problems is that we can express the agent’s goal as the cumulative rewards throughout the agent–environment interaction. For example, if we consider the goal of the maintenance management to be the asset life extension, then we can reward the agent by a positive value for each year that the asset does not fail. Figure 5 depicts the general framework of the RL agent and environment interaction.

**Figure 6:**
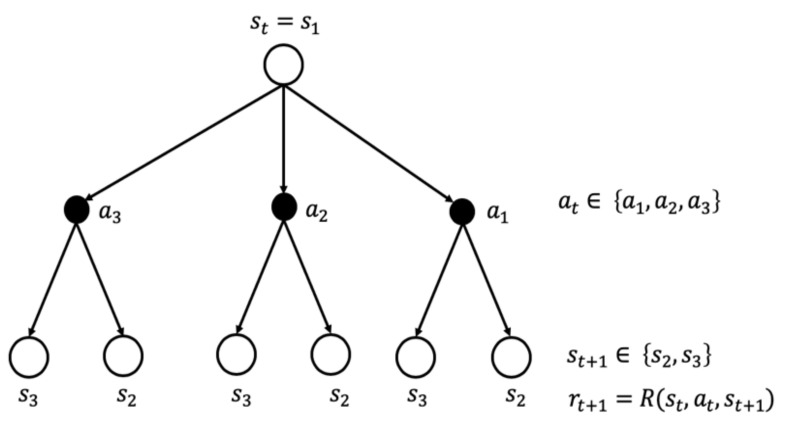
Transition between two consecutive time steps for a simple Markov Decision Process (MDP).

A Markov Decision Process (MDP) formally describes a framework for RL. The MDP is a mathematical abstraction that is defined by a tuple 〈S,A,R,T〉 in which S is a set of states, A is a set of actions, R is a reward function, and T is a transition probability function [39]. In a general condition-based maintenance management setting, the state is the health condition of the asset. In each health state of the asset s ϵ S, the decision-maker takes a maintenance action from the set of possible actions at that state a ϵ A_s_. Upon taking action a, the asset health condition will change and over time reach a new state  s ′, derived from the transition probability function $T(s^{\prime }|s,a)$, and consequently the agent receives a reward r derived from the reward function $R\left(s,a,s^{\prime }\right)$. Figure 6 shows the transition in two consecutive time steps for a simple MDP. The agent starts at time t from state $s_{1}$ and has three choice of actions $a_{t}\;\epsilon \;\left\{a_{1},a_{2},\;a_{3}\right\}$. Depending on the action it takes, the agent lands either on $s_{1}$ or $s_{2}$ according to the transition probability $P(s_{t+1}|s_{t},a_{t})$ and receives the reward $r_{t+1}=R\left(s_{t},a_{t},s_{t+1}\right)$.

Often MDPs are used as discounted MDPs (DMDPs) for RL problems. A DMPD is defined with the same tuple except that it has one extra element which is a discount factor γ. By discounting future rewards with γ, we can determine the present value of future rewards. Without loss of generality, here we consider the asset to be an underground dry natural gas pipeline and the degradation mechanism to be the internal corrosion. However, we want to emphasize that the same approach is applicable to other assets under single or multiple co-existing degradations. We focus on one segment of the pipeline and we assume it is in operation under stochastic environmental and operational parameters. During the operation, the pipeline segment gradually builds up corrosion and degrades. In the absence of proper timely maintenance actions, the corroding pipeline can put the whole pipeline system at the risk of highly hazardous failures, namely leakage and rupture.

### 3.2. The RL Agent Development

This section discusses the development of the proposed decision-maker, which is responsible for maintenance scheduling. The heart of the maintenance scheduler is an RL algorithm, called the agent, that makes the maintenance decisions based on its experience from interacting with the corrosive pipeline. The first step to frame the pipeline’s corrosion maintenance as a sequential decision-making problem is to define it in an MDP format. As stated before, an MDP is the mathematical formulization of RL, and it consists of a state space, an action space, a reward function, and a state transition function. In the following, each one of these elements is described except for the transition function because the sequential decision-making technique employed in this research is model-free RL; i.e., it does not need explicit knowledge about the underlying pipe model that is used to simulate the degradation of the system and it only requires some signals from the state of the pipeline system.

#### 3.2.1. State Space

The states in an MDP must be defined in a way that allows them to have the Markov property; i.e., the future state depends only on the current state and the current action. In other words, the state is a concise summary of the environment’s history that includes all the necessary information to predict the next state given the action. Because the scope of this research is the corrosion of the pipeline, the state definition should include all the essential information to predict the next corrosion status given the action. Therefore, we initially design the state definition to include the depth and length of the corrosion. However, instead of directly taking the value of the depth and length, the max-normalized version of them is considered to remove the agent’s dependency on the pipeline’s parameters. Equations (15) and (16) define the corrosion depth and length as utilized in the pipeline’s health state definition where the maximum corrosion depth is the wall thickness and the maximum corrosion length is estimated by running the model for 40 years without maintenance.

(15) $$CDP=\frac{corrosion\;depth}{maximum\;corrosion\;depth}$$

(16) $$CLP=\frac{corrosion\;length}{maximum\;corrosion\;length}$$

Representing the corrosion state with only the depth and length is not accurate because the next state of the corrosion is not predictable without knowing the rate of the corrosion degradation. Therefore, the corrosion rate is added to the state variables. We assume the agent’s access to the state variables is feasible only through monthly inspections of the corrosion depth and length and, therefore, the corrosion rate is derived by comparing the current month’s corrosion with the previous month’s corrosion. Since corrosion is a slow and gradual process, the agent does not need high precision in state representation. Thus, we discretize the state variables into 24 bins as shown in Table 5. The corrosion rate is represented (CRP) as a binary variable with a value of 0 when there is no corrosion aggravation and 1 when the corrosion exacerbates. Equation (17) formulates the corrosion rate presence as the third state variable.

(17) $$CRP=\left\{\begin{array}{c} 0\;\;\;\;\;if\;\;\;CDP_{t-1}=CDP_{t}\;\;\;and\;\;\;\;CLP_{t-1}=CLP_{t}\;\;\;\\ 1\;\;\;\;\;if\;\;\;CDP_{t-1}\neq CDP_{t}\;\;\;\;or\;\;\;\;\;\;CLP_{t-1}\neq CLP_{t}\; \end{array}\right.$$

#### 3.2.2. Action Space

The action space is defined by available actions that the maintenance scheduler can take in response of the system state. As presented in the maintenance actions section, the benchmark provides the scheduler with five actions. Therefore, a discrete action space of size 5 is considered for the agent as follows,

(18) $$\left\{\mathrm{Do}\;\mathrm{nothing},\;\mathrm{Batch}\;\mathrm{corrosion}\;\mathrm{inhibitor},\;\mathrm{Internal}\;\mathrm{coating},\;\mathrm{cleaning}\;\mathrm{pigging},\;\mathrm{Replacment}\right\}.$$

#### 3.2.3. Reward Function

The reward is a feedback signal from the environment to the agent that reflects the aptness of the RL agent’s actions from the environment’s prospect. In real-world RL problems, the reward function is usually a handcrafted variable by an expert that is derived from the environment’s conditions. Assigning the reward function is a longtime challenge in RL [40]. In this research, the test bench provides the agent with approximated cost values on a monthly basis. Therefore, the agent can use the negative cost as the reward signal. As stated in Section 2, the total cost signal is the algebraic summation of the three types of costs, each of which reflects one of the expectations from the maintenance scheduler. Equation (19) formulates the reward signal. The bold italic terms are binary variables that represent the presence or absence of the term. For example, if pigging is the order of the maintenance in the past month, then the “pigging” value is set to 1; otherwise, it is 0. “∨” signifies the Boolean or.

(19) $$\begin{array}{ll} total\;monthly\;cost & \\ & =\left[480\times \mathrm{leak}+1760\times \mathrm{burst}\right]\\ & +[3.5\times \mathrm{pigging}+13\times \mathrm{inhibitor}+80\times \mathrm{coating}]\\ & +160\times \mathrm{replacement}]+[\left(1-\left(\mathrm{leak}\vee \mathrm{burst}\right)\right)\times \left(-7\right)] \end{array}$$

#### 3.2.4. The Learning Algorithm

Many RL algorithms have been proposed since the rise of RL in the early 1980s that can broadly be classified into two main categories: tabular solution methods and approximate solution methods [6]. Tabular solution methods are applicable to problems with small action and state spaces while approximate solution methods are pertinent for much larger problems [41]. The test bench presented in this research has finite action and state spaces that can be represented in arrays and tables. Accordingly, tabular solution methods are chosen over the approximate solution methods. Besides, the applied algorithms are model-free because the approach of this research is entirely data-driven; i.e., the agent treats the model as a black box that mimics a real pipeline and emits the required data for the learning process.

Temporal difference (TD) learning methods are the most common learning methods among the model-free, tabular RL solutions. The idea of TD learning is to update the current estimate of a variable toward a better estimate of that given the information gained in the current time step. Many algorithms are devised around the TD concept in RL but the most popular one is Q-learning [42], which has been applied to a wide range of problems spanning from healthcare [43] to financial markets [44]. The popularity of Q-learning is due to its simplicity in formulation, implementation, and analysis as well as having reasonable computation and memory costs. In this research, we applied the Q-learning algorithm to the problem of pipeline optimal corrosion maintenance management.

Q-learning aims at learning the optimal action-value functions (also known as the Q-value) to derive the optimal maintenance management policy. As defined by [6], the action-value function, $Q^{\pi }\left(s,a\right)$, is a function that estimates the worthiness of each action at each state and is defined as the cumulative discounted future rewards that can be expected if the agent starts from state s then performs action a and follows the policy π:

(20) $$Q^{\pi }\left(s,a\right)=\;E\left[\overset{episode\;length}{\underset{k=0}{\sum }}\gamma^{k}r_{t+k+1}|s_{t}=s,\;a_{t}=a\right]$$

Patently, the optimal policy is to always choose the action that corresponds to maximum worthiness; i.e., maximum Q-values. It should be noted that any data-driven RL algorithm can only estimate the Q-values according to its experiences, and as it interacts more with the environment, it can arrive at a better estimate of the Q-values. Therefore, choosing the optimal action according to the current estimate of Q-values exploits the current knowledge about the actions’ worthiness. However, there might be other actions with higher actual value functions that are not chosen because the current estimates of the value functions are not accurate. Therefore, the agent needs to balance between exploiting the current knowledge about the best actions and exploring the other actions to gain more accurate estimates. This is known as the exploration–exploitation trade-off [41].

**Figure 7:**
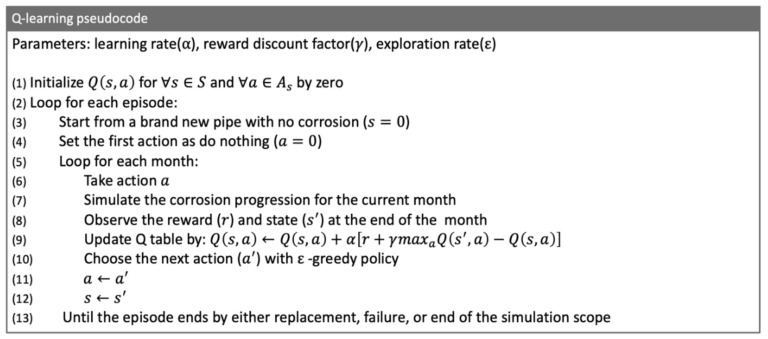
Pseudocode for the implemented Q-learning algorithm.

The pseudocode of the implemented Q-learning algorithm for optimal pipeline corrosion maintenance management is shown in Figure 7. This algorithm is mainly borrowed from [6] and adopted to the pipeline’s corrosion maintenance problem. It consists of one main loop over each episode. In the pipeline corrosion environment, a full episode is defined as the agent that starts with an intact new pipeline and performs a sequence of actions until it either reaches the end of the simulation scope or the pipeline is replaced by taking a replacement action, or a leakage or a rupture failure happens. The exploration–exploitation dilemma is addressed at line (10) by means of an ε-greedy policy; i.e., the agent picks the next action to be the current best action ($(a^{\prime }=argmax_{a}Q\left(s,a\right)$) with probability $\left(1-\varepsilon \right)$ and with ε probability randomly choosing among suboptimal actions.

The problem with the basic ε-greedy policy is that it lacks the ability to adapt as the agent state of knowledge about the actions and the environment improves. Therefore, it always explores the action space with the same rate. If the value of the ε is chosen to be small, the agent does not explore enough and usually sticks to a suboptimal policy; e.g., performs coating when there is small corrosion in the pipeline. If the value of ε is large, the agent keeps exploring even when it is mature, and it should be confident about the best policy. Thus, the decaying ε-greedy policy is implemented as a remedy for this issue. In decaying ε-greedy, the agent starts with a large exploration rate and reduces the ε with each learning iteration. Consequently, the agent leans more towards exploring the actions at the beginning of the learning, and as the learning progresses and the agent get more mature, it favors exploiting its current knowledge about the actions [45]. The decaying rule implemented in this study is given in Equation (21), where $\max\left(\epsilon \right)$ is the maximum exploration rate, $\max\left(\epsilon \right)$ is the minimum allowable exploration rate, and dϵ is the exploration decay rate.

(21) $$\epsilon =\min\left(\epsilon \right)+\left(\max\left(\epsilon \right)-\min\left(\epsilon \right)\right)e^{d\epsilon \times iteration}$$

## 4. Evaluation Methodology

In this section, we describe how to quantitatively evaluate the performance of the proposed RL maintenance scheduler in achieving its three goals. First, a set of performance metrics are proposed, and then, to acquire a baseline for performance comparison, a periodic maintenance scheduler is introduced. Later in the results and discussion section, the performance of the RL condition-based scheduler is compared with the periodic scheduler based on the proposed metrics. Finally, the results of the sensitivity analysis are discussed to study the influence of the model parameters.

### 4.1. The Maintenance Scheduler Performance Evaluation

The maintenance scheduler has three objectives, namely avoiding catastrophic failures, extending the asset’s life, and reducing the maintenance cost. A metric of performance is introduced for each one of the objectives to evaluate the agent’s performance in accomplishing its goals quantitatively.

1. Number of failures in n full episodes runs of the test bench.
2. Average life length in the unit of month over n full episodes runs of the test bench.
3. Average monthly cost (excluding the life extension reward) over n full episodes runs of the test bench.

The performance metrics are average values in multiple runs to address the randomness of the test bench due to the stochasticity in the corrosion model’s input variables; e.g., environmental and operating parameters. In this study, we choose n=20. Therefore, 20 full pipeline life episodes are simulated by the test bench while the maintenance decision-maker instructs the monthly maintenance orders. A full episode is defined by starting from a new pipeline and simulating until either reaching the maximum number of steps or entering the terminal state (i.e., the replacement of the pipeline or the occurrence of leakage or rupture).

A periodic maintenance policy is the most common maintenance practice in the oil and gas industry. Therefore, the performance of the periodic maintenance policy serves as the baseline performance for evaluating the RL agent’s performance. A periodic maintenance strategy carries out the maintenance actions in fixed periods. To account for the variations of the periodic maintenance policy given that each pipeline operator has their preference on the period of each maintenance action, multiple evaluations by the periodic maintenance policy with different scenarios are performed. The considered scenarios are listed in Table 6.

### 4.2. Sensitivity Analysis

The test bench presented in this paper is a valuable tool for when the pipeline operators and decision-makers want to assess their maintenance policy, and the learning algorithm of Section 3.2.4 is beneficial when they want to consult an AI technique for making an optimal maintenance management strategy. However, it is crucial to tune the test bench and adjust the models’ parameters to each specific pipeline system. Hence, in this section, we introduce sensitivity scenarios to study the influence of some of the test bench’s parameters, namely, the maintenance actions’ effective life and the life extension reward, on the RL agent’s performance.

Five scenarios of sensitivity analysis are designed and applied to the proposed methodology. The Q-learning agent is trained for each sensitivity scenario separately, and afterward, the resulting policy is evaluated by the same approach as explained in the previous subsection. The parameters of each scenario are listed in Table 7. The value of the life extension reward and the effective lifetime of maintenance actions are changed one at a time. Therefore, the results reflect the influence of each variable independent from the others. The scenario that holds the former life extension reward and maintenances’ lifetimes parameters, presented in Table 3 and Table 4, is addressed as the reference for the sensitivity scenarios.

## 5. Results and Discussion

The RL agent’s performance in a maintenance scheduling task of a corrosive pipeline in the test bench is evaluated according to the previous section, and this section presents the evaluation results. In the first step, the performance of the 12 periodic maintenance scenarios (shown in Table 6) are evaluated to obtain a baseline performance. Afterward, the performance of the trained RL agent is compared with the baseline. Finally, the results of the RL agent sensitivity analysis on both maintenances’ parameters and the life extension reward are discussed.

### 5.1. Establishing the Baseline: Periodic Maintenance

The baseline enables the quantitative analysis of the RL agent’s performance because we need a baseline performance to compare the agent’s performance against that. In this research, we investigated various scenarios of the periodic maintenance policy to define a baseline (see Table 6). For each periodic maintenance scenario, 20 full pipeline life episodes were simulated by the test bench while the maintenance actions were instructed according to each specific scenario.

Table 8 shows the results in the form of the three performance metrics for the 12 periodic maintenance scenarios. The results show that although none of these periodic maintenance scenarios lead to rupture failures, as many as 9 out of 12 periodic maintenance scenarios encounter leakage failures in some of the 20 evaluation episodes, and they are regarded as failure scenarios. On the other hand, scenarios 1, 4, and 7 have no failure in all the 20 evaluation episodes, and they are regarded as successful scenarios.

The best scenario among all the successful ones is scenario 7, which has the highest average lifetime and the lowest average monthly cost. Although it has roughly the same average lifetime and average monthly cost with scenario 10, it has no failure in 20 episodes simulations. Therefore, it is picked as the baseline performance for comparison purposes.

### 5.2. Evaluation of the Proposed Maintenance Scheduler

In the first step, the Q-learning agent is trained in the test bench for 1000 episodes with a length of 40 years. The minimum and maximum exploration rates within the training are set to 0.0005 and 0.8, respectively, and the exploration decay rate is set to 0.002. After the training step, the performance of the fully trained Q-learning agent is evaluated through 20 test episodes with the same performance metrics presented in Section 4. The performance of the Q-learning agent in comparison with the best periodic maintenance policy is shown in Table 9.

According to Table 9, during the evaluation phase the trained RL agent achieved the full-life length in all 20 evaluation episodes without any failure while reducing the cost to less than half of the best periodic maintenance scenario. This result verifies that there is a great potential to reduce the maintenance costs and improve the reliability of the pipeline by deploying an RL approach in maintenance management. RL achieves a superior performance on the grounds of its closed-loop maintenance policy improvement; i.e., the policy is improved by processing the previous maintenance experiences.

**Figure 8:**
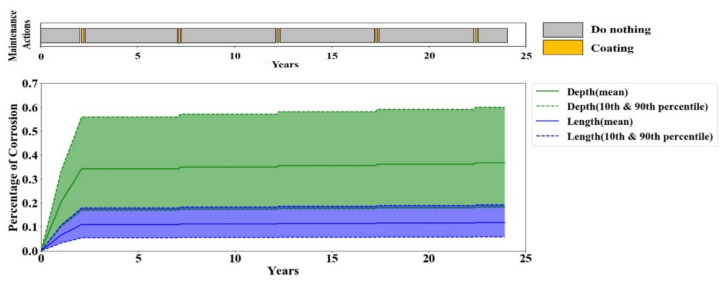
Corrosion depth and length in one full-life evaluation episode by Q-learning.

To further investigate the maintenance policy behavior obtained from Q-learning, the corrosion depth and length in one full-life evaluation episode are plotted in Figure 8. The maintenance policy is derived from the Q-values of the agent after 1000 episodes of training, and the timely maintenance actions are illustrated as a maintenance bar on top of each plot. The results show that the Q-learning tends to perform no maintenance action until the corrosion becomes moderately severe, and then it repetitively performs coating. During the 5-year effective lifetime of the coating, the agent decides to do nothing because there is no progress in corrosion and any maintenance action would have no effective trace on the corrosion. This simple policy is rather obvious to human brains; however, it is a great achievement for the agent because it found this policy while it was blind to any details of the model—e.g., the coating life and cost, etc.

### 5.3. Results of the Sensitivity Analysis

The performances of the fully trained Q-learning agent under the various sensitivity analysis scenarios in comparison with the reference sensitivity scenario are shown in Table 10. According to this table, none of the 20 evaluation episodes in any of the scenarios ended with failure, and all of them survived until the end of the evaluation simulation; i.e., 288 months. This serves as evidence that the Q-learning agent successfully arrives at the maintenance management policies with a very low risk of failure in all the scenarios. However, the average monthly cost varies in each scenario.

**Figure 9:**
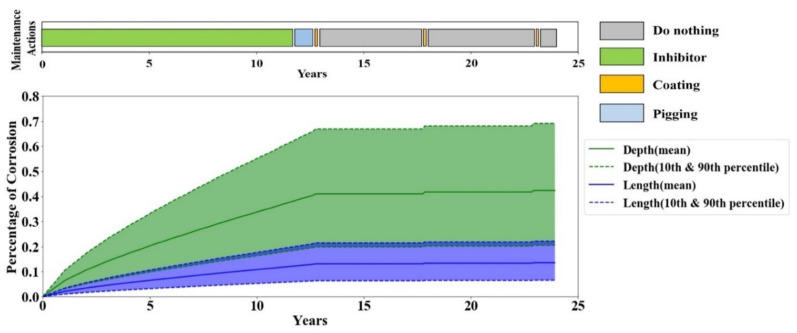
Corrosion depth and length in one full-life evaluation episode for sensitivity scenario one.

In sensitivity scenario one, the life extension reward is reduced from 7 to 1 which would make the agent less eager to leave longer. However, the results show no reduction in the pipeline’s average life length in scenario one. This observation is due to the fact that the only way of terminating the pipeline’s life prior to the end of the simulation is through either of the failures which are followed by very large costs. Hence, although the agent has a lower tendency to extend the pipeline’s life, it still survives until the last step of the simulation solely to avoid the large costs associated with the failures. On the contrary, the average monthly cost of the Q-learning optimal policy in scenario one does not remain the same and escalates to around 1.5 times higher than the reference sensitivity scenario. This can be seen in Figure 9 which shows that the agent trained under scenario one performs inhibitor in the beginning until the corrosion becomes moderately severe, and then pigging is performed for a short period before it repetitively performs coating and does nothing during the lifetime of the coating. Thus, the frequent application of an inhibitor leads to a higher average monthly cost compared to the reference scenario.

**Figure 10:**
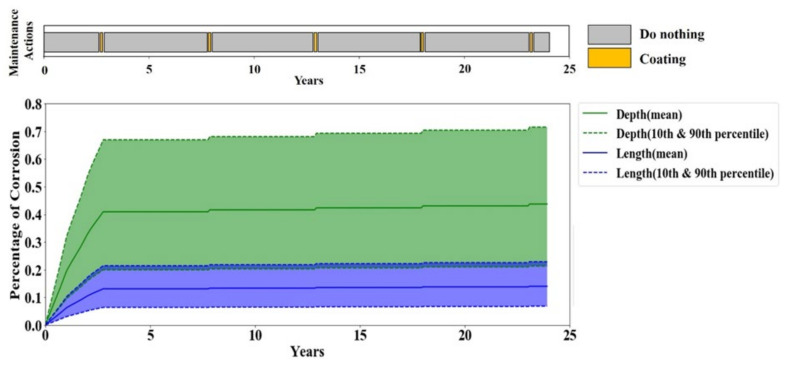
Corrosion depth and length in one 24-year full life evaluation episode for sensitivity scenario two.

In scenario two, the life extension reward is increased to 9, which does not have any effect on the Q-learning final policy in terms of life length and average monthly cost, as shown in Table 10. In addition, the corrosion depth and length progression and timely maintenance actions in scenario two, Figure 10, are identical to the reference scenario (see Figure 8) that the agent performs no maintenance action until the corrosion becomes moderately severe, and then it repetitively performs coating and does nothing during the lifetime of the coating. This observation can be related to the fact that the simulation time horizon is not long enough to capture the effect of a higher life extension reward. Therefore, another training and evaluation set with a simulation scope of 60 years is performed only for the second sensitivity scenario.

**Figure 11:**
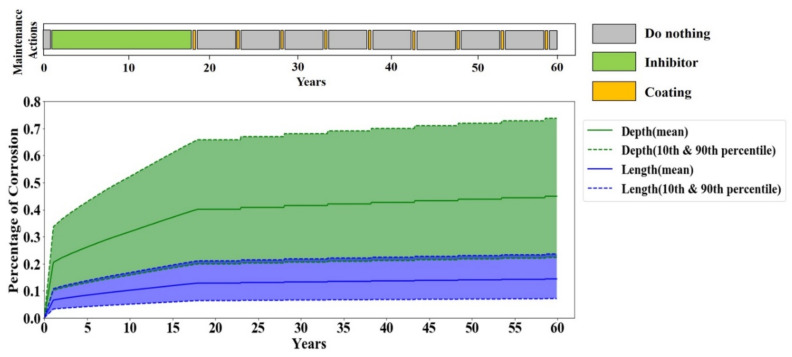
Corrosion depth and length in one 60-year full-life evaluation episode for sensitivity scenario two.

The results of the agent with 60 years scope endorsed that the Q-learning agent can survive until 60 years for all the 20 evaluation episodes. The corrosion depth and length progression and timely maintenance actions for 60 years of evaluation are shown in Figure 11. According to this figure, the Q-learning agent decides to apply inhibitor for the first 18 years of the evaluation period, and afterward it performs coating every five years. Comparing Figure 10 and Figure 11 reveals that applying the inhibitor in the first 18 years is what assists the Q-learning agent to survive up to 60 years. The reason is if the Q-learning agent took the same policy as Figure 10 for the 60 years scope, the corrosion would have reached 100%, which would have certainly resulted in failure.

**Figure 12:**
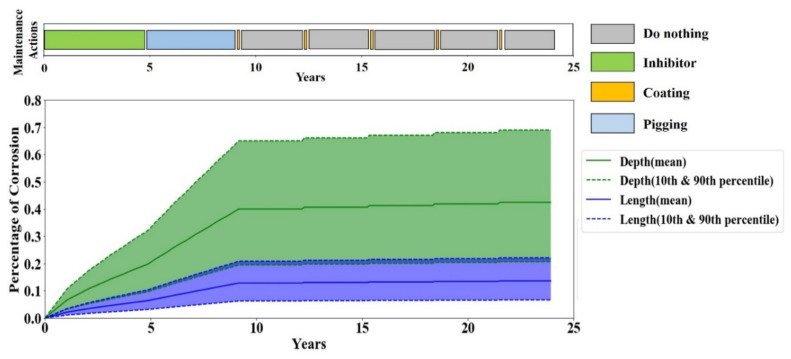
Corrosion depth and length in one full life evaluation episode for sensitivity scenario three.

In the third scenario, the coating life decreased from 5 years to 3 years and, therefore, it is expected that the agent chooses coating less often because it becomes less cost-effective. Figure 12 endorses this proposition. As it can be inferred from Figure 12, the agent chooses inhibitor and pigging until around year 10; then it starts switching between doing nothing and coating. Although the coating is favored less, the agent still prefers coating over other actions when the corrosion becomes more severe because the coating is still the most conservative maintenance action that forces the corrosion rate to zero in the time it is effective. Since the chances of failure are higher when the corrosion is more severe, the agent would not risk taking any action other than the most conservative action—i.e., coating.

By comparing the frequency of applying the coating in the third scenario (see Figure 12) with the reference scenario (see Figure 8), it is found that the coating is performed every three years in the third scenario, while it is executed every five years in the reference scenario. This serves as a confirmation that the Q-learning agent closely follows the coating life such that as soon as the coating life comes to the end, the agent applies another coating action. In addition, a more frequent need for an application a coat is the main contributor to a higher average monthly cost in scenario three.

**Figure 13:**
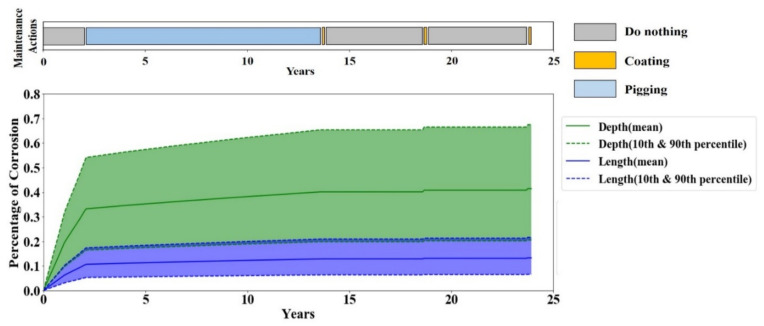
Corrosion depth and length in one full life evaluation episode for sensitivity scenario four.

The fourth scenario is designed to study the sensitivity of the Q-learning optimal policy to the effective life of the pigging. According to Figure 13, pigging is chosen more often when its effective life is increased from 2 weeks to 1 month. The reason for this is that the agent spends the same cost while it experiences a longer halt in corrosion progression which makes pigging two times more cost-effective than before. Despite the fact that the pigging is taken more often, the agent repeatedly prefers to perform coating when the corrosion becomes more severe. This behavior is observed in the third scenario as well, and it can be related to the fact that the coating is the most conservative action of all.

Moreover, scenario four is the only scenario with a lower average monthly cost than the reference scenario. The lower average monthly cost is due to performing the pigging instead of the coating during the first half of the pipeline’s life. It might be expected that the average monthly cost is drastically lower in the fourth scenario comparing to the reference scenario because the pigging cost is 23 times less than the coating cost, according to Table 5. However, it is worth mentioning that although the pigging cost is much smaller, it needs to be repeated every month while coating is carried out once every five years. Hence, the cumulative pigging cost is comparable to the coating cost.

**Figure 14:**
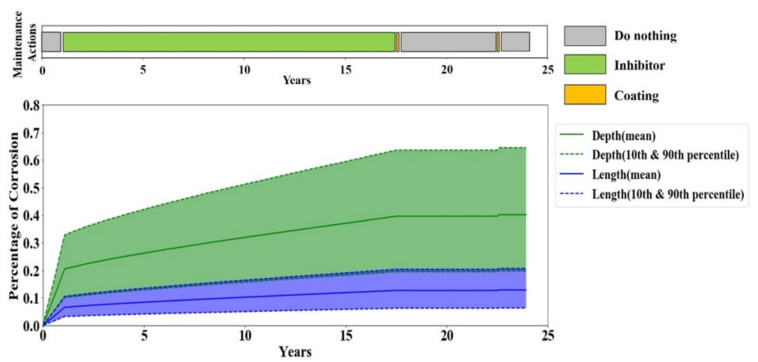
Corrosion depth and length in one full life evaluation episode for sensitivity scenario five.

The last sensitivity analysis scenario is designed to study the consequence of increasing the inhibitor effectiveness by prolonging its life to 4 months. As Figure 14 shows, the result is that the agent selects inhibitor more frequently such that it is chosen as the optimal action in the first 17 years. Then, similar to scenario three and four, as the corrosion reaches more severe states, the agent favors coating over the inhibitor because more severe corrosion is associated with a higher likelihood of failure, and the agent has the tendency to act very conservative in these states.

## 6. Conclusions

This paper presented a test bench and a methodology for addressing the problem of optimizing maintenance management of a dry gas pipeline by RL decision-making approaches. Despite the great recent achievements of RL and its promising capabilities to improve asset integrity management by leveraging the enormous amount of IoT data, its application to pipeline integrity management is very limited. The proposed test bench is an effort to fill this gap via facilitating the development of RL methodologies in pipeline integrity management without risking the real system. The test bench is a physics-based corrosion degradation model that could interact with a decision-maker agent and adjust itself to the maintenance orders conducted by the decision-maker. Accordingly, it provides a testbed for assessing various corrosion maintenance policies including but not limited to any RL-based maintenance policy, condition-based maintenance policy, periodic maintenance policy and corrective maintenance policy.

Furthermore, this paper proposed a data-driven, model-free RL algorithm that performs condition-based maintenance management. The proposed algorithm is trained and tested through the test bench, and the results showed a 58% reduction in maintenance costs while improving the reliability of the pipeline compared to the periodic maintenance regime; i.e., no failure happened under the proposed condition-based maintenance policy. These promising results encourage further investigations into applying data-driven RL approaches to pipeline integrity management. In continuation of this research, the authors are working on advancing the RL agent to overcome the limitations of the current agent, such as finite and discreet state and action spaces. More specifically, the authors are working on development of a data-efficient and trustable deep RL solution that would be a competent decision-maker to deal with real pipeline systems. Accordingly, we have been in continuous consultation with a number of major oil and gas companies on modeling aspects and assumptions of operational parameters and maintenance strategies.

## Figures and Tables

**Table 1:** Operating parameters of the gas pipeline system.

Variables	Type	Mean	COV
T (K)	Normal	308	0.10
P (bar)	Lognormal	56.6	0.15
pH_2_S (bar)	Lognormal	3	0.15
pCO_2_ (bar)	Lognormal	4	0.15
V (m/s)	Lognormal	5	0.10
pH	Lognormal	4	0.06
Cl^−^ (ppm)	Lognormal	150,000	0.15
SO_4_^2−^ (ppm)	Lognormal	2000	0.15
		Upper limit	Lower limit
R_solid_	Uniform	1	0.5

**Table 2:** The maintenance scheduler set of maintenance actions.

Actions	Descriptions	Comments
Do nothing	No mitigation is done	The corrosion proceeds
		Corrosion inhibitor is added from the inlet of the pipeline
Batch corrosion inhibitor	A chemical that adsorbs onto the metal surface and reacts with it to form a protective film	Corrosion rate drop is based on the inhibitor efficiency Effective only within its lifetime
Internal coating	An artificial coating that isolates the pipe from the corrosive environment and prevents water from reaching the pipe surface	No corrosion propagation during its lifetime
Cleaning pigging	A gadget that effectively cleans up liquids, corrosive solids and debris	No corrosion propagation during its lifetime
Replacement	Replace the corroded segment with a new one	Renew corrosive environment No more corrosion defects

**Table 3:** Discount factors and lifetime of maintenance actions.

Actions	Discount Factors	Lifetime
Do nothing	$df_{do\_nothing}=1$	-
Batch Corrosion Inhibition	$df_{inhibitor\;}=1-0.9487e^{-0.023\times inhibitor\_lifetime}$	1 month [33]
Internal coating	$df_{coating}=0$	5 years [24]
Pigging	$df_{pigging}=0$	2 weeks [33]

**Table 4:** Values of reward functions for different types of costs in the unit of USD 10,000.

Type	Cases	Value
Maintenance cost	Do nothing	0
	Pigging	−3.5
	Inhibitor	−13
	Coating	−80
	Replacement	−160
Failure cost	Leakage failure	−160 × 3 = −480
	Rupture failure	−160 − 1600 = −1760
Life extension reward	Life extension reward per month	+7

**Table 5:** Discretized representation of the state space.

CRP	CDP	0–20%	20–40%	40–60%	60–100%
	CLP				
0	0–33%	0	1	2	3
0	33–66%	4	5	6	7
0	66–100 +%	8	9	10	11
1	0–33%	12	13	14	15
1	33–66%	16	17	18	19
1	66–100 +%	20	21	22	23

**Table 6:** Different scenarios of periodic maintenance policy (period in months).

Scenario No.	Maintenance Actions Period in Months			
	Inhibitor	Coating	Pigging	Replacement
1	1	120	1	288
2	2	120	1	288
3	3	120	1	288
4	1	180	1	288
5	2	180	1	288
6	3	180	1	288
7	1	120	2	288
8	2	120	2	288
9	3	120	2	288
10	1	180	2	288
11	2	180	2	288
12	3	180	2	288

**Table 7:** Different scenarios of RL agent sensitivity analysis.

Sensitivity Analysis Scenario	Life Extension Reward (USD 10,000)	Coating Effective Lifetime (Months)	Pigging Effective Lifetime (Months)	Inhibitor Effective Lifetime (Months)
Reference	+7	60	0.5	1
1	+1	60	0.5	1
2	+9	60	0.5	1
3	+7	36	0.5	1
4	+7	60	1	1
5	+7	60	0.5	4

**Table 8:** Performance summary for the 12 periodic maintenance scenarios in 20 evaluation episodes.

Periodic Policy Scenario	Number of Rupture Failures	Number of Leakage Failures	Average Life Length	Average Monthly Cost
1	0	0	288.0	13.40
2	0	5	273.0	14.38
3	0	20	216.5	33.28
4	0	0	288.0	12.43
5	0	20	215.6	34.14
6	0	20	154.1	42.45
7	0	0	288.0	11.65
8	0	20	111.9	55.99
9	0	20	113.6	53.91
10	0	1	287.8	11.66
11	0	20	105.3	58.87
12	0	20	106.6	56.87

**Table 9:** Performance comparison of the fully trained Q-learning agent and the best periodic maintenance policy during the evaluation phase in 20 evaluation episodes.

Policy From	Number of Rupture Failures	Number of	Average	Average Monthly Cost
		Leakage Failures	Life Length	
Q-learning	0	0	288	4.86
Periodic Scenario #7	0	0	288	11.65

**Table 10:** Performance summary for the sensitivity analysis scenarios in comparison with the reference sensitivity scenario in 20 episodes.

Sensitivity	Average Number of Rupture Failures	Average Number	Average	Average Monthly Cost
Scenario		of Leakage Failures	Life Length	
Reference	0	0	288	4.86
1	0	0	288	7.03
2	0	0	288	4.86
3	0	0	288	7.62
4	0	0	288	4.34
5	0	0	288	5.03
